# Green finance improves enterprises’ environmental, social and governance performance: A two-dimensional perspective based on external financing capability and internal technological innovation

**DOI:** 10.1371/journal.pone.0302198

**Published:** 2024-04-17

**Authors:** Hongfeng Zhang, Shuying Wei

**Affiliations:** 1 School of Public Administration and Policy, Shandong University of Finance and Economics, Jinan, China; 2 School of Economics, Shandong University of Finance and Economics, Jinan, China; University of Almeria: Universidad de Almeria, SPAIN

## Abstract

This paper takes the establishment of the Green Financial Reform and Innovation Pilot Zone (GFPZ) in 2017 as a natural experiment, adopts the data of a-share industrial listed enterprises in Shanghai and Shenzhen from 2010 to 2020, and utilizes the difference-in-differences (DID) method to carry out empirical tests. The results show that (1) GFPZ policy significantly improves the environmental, social, and governance (ESG) performance of enterprises, and the positive effect is mainly realized by improving the external financing ability and green-technology innovation level of enterprises. (2) There is heterogeneity in the impact of GFPZ policy on the ESG performance of firms with different equity natures and internal control levels. (3) Green finance promotes active corporate social responsibility, and it can further improve environmental governance in the regions where it operates. This paper provides a useful supplement to the comprehensive understanding of green-finance policy effects and ESG impact factors, and it is of great significance in mitigating the negative environmental and social externalities caused by the excessive pursuit of economic benefits by enterprises.

## 1. Introduction

Sustainable development has once again become an active topic of discussion internationally due to the multiple pressures of extreme global climate change, increasing shortage of energy resources, ecological and environmental assistance, and prominent social problems. In recent years, China’s economy has developed rapidly, creating the world-renowned China Miracle, but the crude production model of traditional industrial enterprises has brought about massive energy consumption and serious environmental pollution, and the world’s remarkable economic growth has been accompanied by a huge cost in resources and to the environment [1]. Scholars have pointed out that, as the main body of the market, the behavioral choices of enterprises ultimately determine the quality of economic development, and that enterprises should safeguard social and ecological interests while pursuing economic interests and should reduce negative impacts on the environment and society at all of the stages of production and operation [2, 3]. Enterprise attaches great importance to environmental, social, and governance (ESG) standards, which have a positive effect on promoting sustainable economic development [4, 5]. Therefore, in the face of multiple pressures on energy and the environment, incentivizing enterprises to increase their environmental awareness and improve their ESG performance is an effective path to achieving sustainable economic development.

Environmental resources have public attributes, and the costs and benefits of corporate environmental social responsibility are usually externalities, so enterprises that seek to maximize their profits lack sufficient incentives to actively undertake environmental social responsibility. China has introduced a series of institutional innovations and policy tools, such as environmental tax reform, civilized city building, and green finance, in order to raise corporate environmental awareness and encourage companies to take on more social and environmental responsibility. Among them, the reform of environmental tax greening can motivate enterprises to pay attention to environmental issues, thus encouraging enterprises to undertake environmental and social responsibility [6]. Moreover, the construction of civilized cities can help to enhance the government’s environmental regulation, optimize the quality of corporate executives, and improve the social-credit environment, which is conducive to the enhancement of the undertaking of corporate social responsibility [7]. Green finance is a great change in the financial system to address environmental challenges, aiming to intervene in directing the flow of funds by changing the incentives for resource allocation and thus promoting sustainable economic development [8–10]. However, in concrete practice, can green-finance policies effectively incentivize enterprises to actively undertake social and environmental responsibilities? What are the influence mechanisms involved? These questions are important theoretical and practical issues in seeking the coordinated development of the environment and economy in the new era. This paper explores the impact effect and mechanism of green finance on enterprise ESG performance, which can make a marginal contribution to a more comprehensive understanding of the green-finance policy effect, incentivize enterprises to actively undertake environmental and social responsibility, and help the economy achieve sustainable development.

Using the DID model, this study takes the exogenous policy-shock event of the GFPZ in 2017 as a quasi-natural experiment, which greatly avoids the endogeneity problem, and it explores the impacts of green-finance policies on enterprise ESG performance and the mechanism of its role, which has the following marginal contributions: First, it empirically tests that the GFPZ policy has a significant positive impact on enterprise ESG performance, and that this impact has obvious heterogeneity in enterprises with different property-rights natures and different internal control quality, which provides empirical evidence from the perspective of green-finance pilot policy for the exploration of the relationship between green finance and enterprise social responsibility, and it further enriches the theoretical research in the field of green-finance policy-effect assessment. Second, based on the two-dimensional perspective of external financing ability and internal technological innovation, it analyzes in depth the intrinsic mechanism of the role of green finance on the ESG performance of enterprises, which further complements the research content in the field of CSR influencing factors and provides path references to incentivize enterprises to actively undertake environmental and social responsibility. Third, additional research reveals that the incentive effect of green-finance policies on enterprise ESG performance can effectively improve the level of environmental governance in the region where the enterprise is located, with favorable environmental benefits. The findings of the study provide policy insights to assist in addressing key issues affecting sustainable development, such as environmental pollution and ecological damage.

The remainder of the paper is arranged as follows. Section 2 summarizes previous research and points out the limitations of existing research, Section 3 briefly describes the institutional context and sets out the research hypotheses, Section 4 describes the empirical model and the data used in this study. Section 5 presents and analyzes the empirical results. Finally, Section 6 concludes the paper and presents some policy implications.

## 2. Literature review

The literature related to this study consists of three main sections, namely, enterprise social responsibility, the impact of green-finance policies on enterprises, and the relationship between green finance and enterprise environmental and social responsibility.

### 2.1 Literature related to enterprise social responsibility

There has been a long history of discussion of enterprise social responsibility in academia [11]. Enterprise social responsibility refers to the goal of enterprises to maintain and enhance the social good and to the goal of maximizing profits when producing [12]. Traditional theories of business and corporate law aim to maximize corporate profits and shareholder profits as the only goal of business; however, advocates of the concept of corporate social responsibility have suggested that companies should not only make money for shareholders but also provide stable jobs for employees, offer high-quality products for consumers, and improve the welfare of society [13]. Since then, with the aggravation of global environmental degradation and the rise of the green concept, based on the basic idea of building a harmonious relationship between enterprises and society, minimizing the negative impacts of production and operation activities on the environment and actively participating in environmental protection have become the core contents of corporate social responsibility [14]. On this basis, the ESG concept has been proposed and gradually become a key indicator for measuring the nonfinancial performance and sustainability of enterprises [15–17]. ESG refers to the concept that enterprises not only have to consider economic returns but also pay attention to the environmental, social, and governance aspects of their performance. Specifically, environmental means that firms should improve environmental awareness and reduce waste emissions; social means that firms should consider the impact on society, such as on community relations; and governance means that companies should improve their internal governance [18].

Since that time, many scholars have conducted in-depth studies on the impact effects and influencing factors of corporate ESG performance. Regarding the impact effects of corporate ESG performance, existing research has found that firms that improve their ESG performance can reduce the cost of debt [19], improve risk-adjusted returns [20], and reduce exposure to existential risk, thus contributing to the long-term sustainability of the firm [21]. Enterprises can sacrifice some of their financial returns in exchange for more positive environmental or social impacts, which, in turn, can lead to long-term gains [22]. Additionally, enterprise ESG performance affects corporate stock returns, financial performance, market performance, enterprise value, country default risk, and stock-price crash risk to varying degrees [23–28]. Regarding the influencing factors of corporate social responsibility, Baldini find that the political values of institutional shareholders and diversity within the board of directors affect the ESG performance of firms in terms of corporate dimensions [29]. Kim and Kizys also point out that financial-resource conditions and institutional environments, such as political, labor, and cultural environments, also affect firms’ ESG performance [30, 31].

### 2.2 Literature on the impact of green-finance policies on enterprises

Since the launch of green finance, scholars have centered their studies on the impact of green finance on enterprises to carry out research. Thus, the existing literature primarily focuses on green finance and enterprise resource allocation, total factor productivity, enterprise value, technological innovation, and other aspects [32]. Specifically, in terms of resource allocation, Zhang point out that green finance significantly reduces the debt-financing capacity of heavily polluting firms and significantly inhibits their investment behavior [33]. Xu and Li find that green finance has a positive effect on the cost of debt financing for green firms [34]. In terms of productivity, Feng and Liang state that green finance has a negative impact on firms’ total factor productivity [35]. However, Kong argue that green finance increases the total factor productivity of firms in China’s heavily polluting industries [36]. In terms of enterprise value, Yao point out that green credit raises the threshold of enterprise financing by forming credit constraints on heavy polluting enterprises, which, in turn, reduces the financial performance of enterprises [37]. Furthermore, Hu argue that green finance helps enterprises develop in the long run [38]. In terms of technological innovation, Liu point out that green finance can promote the green innovation of polluting enterprises [39]. However, Ling argue that green finance significantly reduces the R&D input and innovation output of enterprises in pollution-intensive industries, and fails to promote technological innovation of enterprises in pollution-intensive industries [40].

### 2.3 The relationship between green finance and enterprise environmental and social responsibility

Song point out that, given their aim to maximize profits, enterprises usually lack sufficient incentives to actively undertake environmental social responsibility and need external constraints to push them to do so [41]. Moreover, by changing the allocation of resources financial institutions can internalize the negative externalities that enterprises may have on the environment and ecology, which can then motivate enterprises to actively fulfill their environmental social responsibility [42]. With the development of green-finance policy and the enhancement of the awareness of environmental social responsibility of enterprises, more scholars have begun to pay attention to the impact of green-finance policy on corporate social responsibility. For example, Qian and Yu argue that green finance acts on corporate ESG performance by affecting corporate financing costs and financing constraints [43]; however, some scholars have pointed out that credit institutions have supervisory and governance functions in environmental protection, and that green credit can incentivize enterprises to undertake environmental responsibility and significantly improve their ESG performance [44–46]. For instance, Wu and Liew prove that the level of green-finance development and corporate ESG performance have a positive correlation through the construction of green-finance development indicators [47]. Sinha hold an opposing view, arguing that the impact of green finance on ESG gradually transforms in a negative direction [48].

Scholars have made many valuable findings around corporate ESG performance and the policy effects of green finance; however, there is still room for improvement in the following aspects: First, scholars have both supporting and opposing views on whether green-finance policies have a positive impact on corporate ESG performance. Also, existing studies have mostly focused on the impact of traditional green-credit policy and green-bond policy on corporate ESG performance, and limited scholars have paid attention to the impact of green-finance pilot policy on corporate ESG performance. Second, the current research on the mechanism of green finance affecting ESG performance is relatively fragmented and not comprehensive enough, and most of it focuses on the external channel represented by financing constraints, so the role of the policy impact channel needs to be further explored. Third, corporate internal control is closely related to corporate organizational decision-making, information communication, and supervision, but limited scholars have paid attention to the impact of corporate internal control quality on the relationship between green finance and corporate ESG performance.

This paper, based on existing research, intends to carry out the following studies: First, we empirically test the impact of green-finance policies on corporate ESG performance to provide empirical evidence for the effect of comprehensive green-finance policies. Second, we explore the mechanism of the role of green-finance policies on corporate ESG performance, to further supplement the research in the field of corporate social-responsibility influencing factors. Last, we further explore the role of the nature of corporate property rights and the level of internal control on the above impact to provide theoretical references for better incentivizing corporations to actively undertake environmental and social responsibility.

## 3. Policy background and research hypotheses

### 3.1 Policy background

Enterprises are important participants and key actors in the implementation of the concept of green development and the realization of sustainable development, as well as direct influencers of green finance. Actively assuming environmental and social responsibility, as a kind of sustainable development orientation in the operation and management of enterprises, is not only conducive to firms’ own long-term development, but it also mitigates the negative externality caused by the over-pursuit of economic benefits, producing a good economic and environmental return [49]. China’s awareness of corporate environmental and social responsibility has been rising in recent years under the guidance of relevant policies, regulations, and external regulatory requirements, among other external drivers [50]. The State-owned Assets Supervision and Administration Commission proposed in 2012 that state-owned enterprises must publish corporate social responsibility reports, and that the fulfillment of social responsibility by enterprises would be included in the state-owned enterprises’ appraisal system, which began to guide the fulfillment of social responsibility by enterprises at the policy level. The China Securities Regulatory Commission revised the Code of Governance for Listed Companies in 2018, in particular introducing the concept of ESG, which is promoted by the international capital market, alongside increasing the content of environmental protection and social responsibility. That same year, the Shanghai Stock Exchange and Shenzhen Stock Exchange issued ESG disclosure guidelines, requiring listed companies to publish ESG-related information on a "disclosure or explanation" basis, and it is becoming a trend for companies to publish social responsibility reports. With the introduction and implementation of "dual carbon" goals, ESG has attracted more attention, and the vice chairman of the China Securities Regulatory Commission said at the annual meeting of the Boao Forum for Asia (BFA) 2022 that China’s next step would be to develop a guideline requiring mandatory ESG disclosure.

At the same time, to better promote ESG practice, China is providing a number of institutional innovations and policy tools, one being green finance. In recent years, green-finance products have emerged continually in China, and related policies and systems have been continually improved. In 2017, the executive meeting of the State Council deployed the construction of Green Finance Reform and Innovation Pilot Zones (GFPZ), deciding to take Guangzhou City in Guangdong Province, Huzhou City and Quzhou City in Zhejiang Province, Guian New District in Guizhou Province, Ganjiang New District in Jiangxi Province, and Hami City, Changji Prefecture, and Karamay City in Xinjiang Uygur Autonomous Region as the first batch of pilot zones, with a focus on promoting green-finance reform and innovation in terms of green-finance standards and innovation in green-finance product services [51]. This is further innovation and exploration in the field of green finance, which can utilize the endowments of the pilot area, such as the level of economic development and industrial structure, to form a "green-finance sample" that can be drawn on and copied, leading to a "Chinese proposal" for green-finance policy and practice in the new era. This paper takes the establishment of GFPZ as a quasi-natural experiment to explore the impact of green-finance pilot policies on enterprise ESG performance.

### 3.2 Research hypotheses

The traditional view is that a contradiction often exists between social responsibility and the goal of profit maximization, whereby social responsibility undermines the profits of investors. The fundamental causes of this contradiction are information asymmetry and externalities. First, under the traditional financial framework, owing to the asymmetry of market information, it is difficult for financial institutions to accurately judge the nonfinancial status of enterprises, such as their environmental contribution when allocating financial resources, and the majority only focus on the financial status of enterprises, thus resulting in enterprises that are less socially responsible receiving more financial resources and enterprises that actively undertake social responsibility in the financial markets not receiving an advantage. Second, for enterprises, taking up environmental and social responsibility has strong positive public-goods externalities, and it is difficult for them to internalize the benefits of externalities, so they lack incentives to take up social responsibility. The establishment of GFPZ has the dual characteristics of mandatory environmental regulation and flexible market means. It not only provides enterprises with preferential policies and a stable financial environment, but it also considers the potential returns, risks, and costs related to environmental conditions when allocating capital resources. The overall aim is to allocate the limited capital resources to projects that are more in line with energy conservation and emission reduction. At this time, enterprises with good ESG performance have a stronger market advantage, and actively pursuing social responsibilities has become profitable. This policy has effectively alleviated the problems of social responsibility externalities as well as environmental information asymmetry and has helped enhance the willingness of enterprises to actively improve their ESG performance. To this end, our first research hypothesis is as follows:

**H1:** The establishment of the GFPZ can improve the enterprise ESG performance in the region.

If enterprises are to carry out environmental governance activities and assume social responsibilities, they need long-term and stable financial support; therefore, having sufficient disposable capital determines whether an enterprise can actually implement ESG practices [52]. The likelihood of implementing ESG activities is lower for enterprises with poorer financial status. For many companies, external financing remains the main source of funding [53]. The establishment of GFPZ has improved the stability of the financial environment in the region, attracted financial institutions to set up branches in the pilot zone, and expanded regional financial supply. Innovating financial products and services and building a green-finance market provides financial service support for enterprise investment and financing, project operation, and risk management, which helps improve the capital turnover efficiency in a region.

Further, green-technology innovation is one of the key ways for enterprises to improve their ESG performance [54]. However, green-technology innovation is characterized by large capital investment and a long profit cycle, which requires long-term and stable financial support in the early stage of R&D. The stable financial environment and innovative financial services provided by the GFPZ provide financial support for enterprises in the region to develop green production technologies. GFPZ simultaneously reduces transaction costs and enhances the attractiveness of innovation elements by reducing governmental intervention and rent-seeking behaviors. Additionally, GFPZ promotes effective and accurate information communication between financial institutions and enterprises, helps reduce the degree of resource mismatch in a region, and encourages capital investment in high-risk but high-value R&D and innovation activities. In conclusion, the following hypotheses are proposed:

**H2a:** GFPZ policy positively affects enterprises’ ESG performance by increasing their external financing capacity.
**H2b:** GFPZ policy positively affects enterprises’ ESG performance by increasing their green-technology innovation level.

In China, there are significant differences between state-owned (SOEs) and non-state-owned (non-SOEs) in various aspects, such as their position in the financial market and their responsiveness to policy. It is generally believed that SOEs are required to assume more environmental responsibility because their actual controller is the state. SOEs have significant advantages regarding financing channels and resource endowments [55], and they are thus able to assume more ESG responsibilities. Non-SOEs have limited financing channels and financial resources [56], and they mostly aim to maximize their profits, resulting in a lack of awareness and ability to actively perform ESG.

The enterprise’s risk assessment, management, and decision-making activities, as well as information communication and oversight, are all closely related to the quality of the enterprise’s internal controls. Enterprises with high-quality internal control can send positive signals to the market and gain a good reputation, thus positively affecting their external financing capabilities. At the same time, the quality of internal control greatly affects the level of corporate governance [57]. High-quality internal control can effectively reduce information asymmetry between corporate executives and other stakeholders, preventing companies from avoiding social responsibility or creating the illusion of active responsibility to maximize shareholders’ profits and deceive stakeholders’ support, and can effectively implementing the "reasonable requirements" imposed on companies by the institutional environment [58]. Thus, the following hypotheses are proposed:

**H3a:** GFPZ policy has a more significant positive effect on the ESG performance of SOEs.
**H3b:** GFPZ policy has a more significant positive effect on the ESG performance of enterprises with high-quality internal control.

## 4. Study design

### 4.1 Model setup

**Fig 1:**
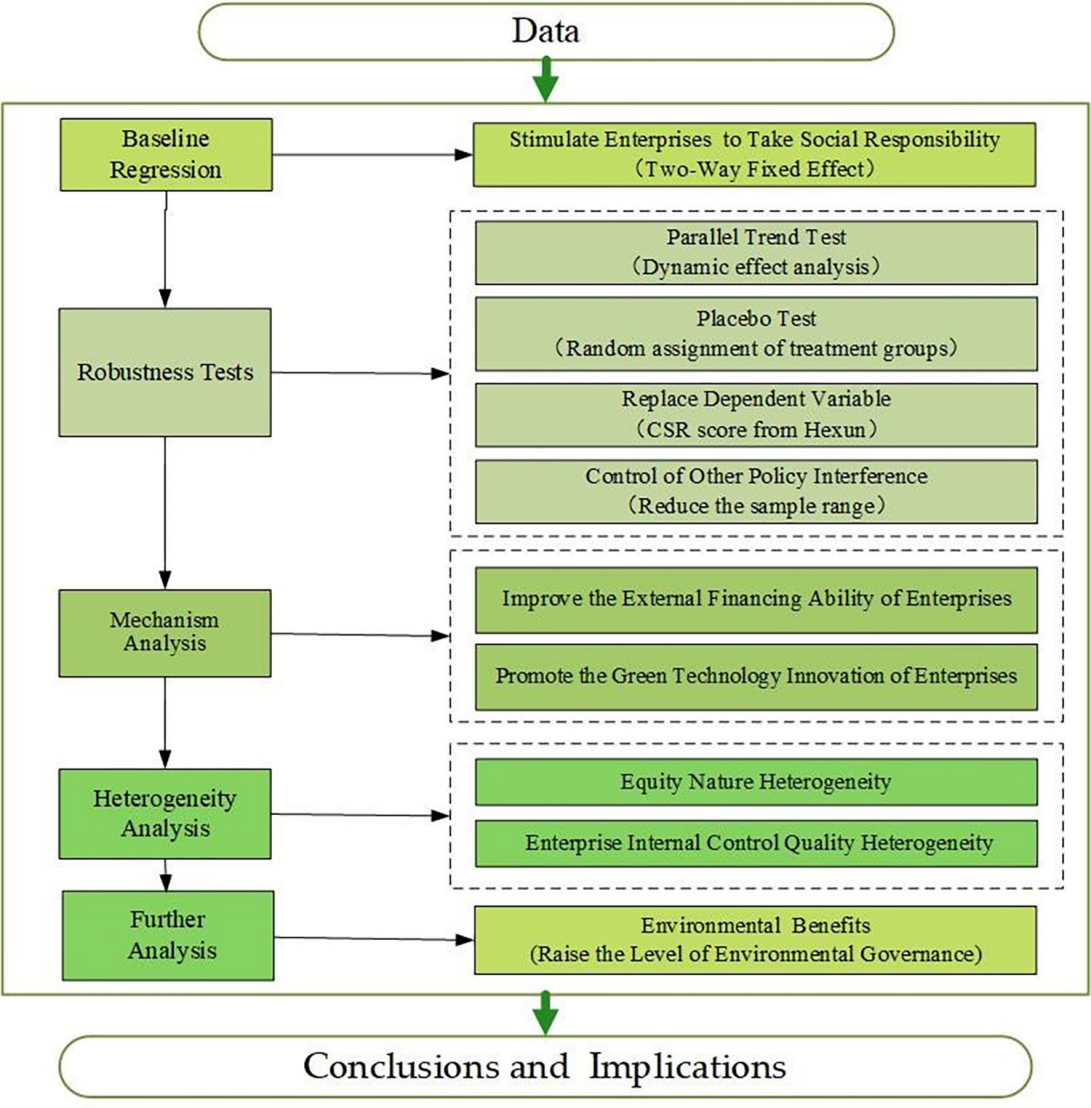
Research framework.

It is clear that current policies, regulations, and external supervision mainly focus on the performance of listed enterprises in performing ESG based the above analysis. For this reason, this paper selects the scope of the research sample in listed enterprises, regards the exogenous policy shock of GFPZ as a quasi-natural experiment, and adopts the DID model to test the impact of this policy on enterprises’ ESG performance. The figure that follows shows the research framework of this paper (**Fig 1**).

The DID model is commonly used to test the effect of policy implementation. First, in contrast to traditional measurement methods, DID uses exogenous events as explanatory variables for research, which largely avoids the problem of endogeneity of explanatory variables, thus allowing for unbiased estimation of policy effects and more accurate policy evaluation.

Second, specifically for the situation studied in this paper, after the implementation of GFPZ policy, changes in the ESG performance of enterprises in the pilot zone primarily originate from the following two aspects: First, even without the establishment of GFPZ, the ESG performance of enterprises may change over time (i.e., the time effect), and consequently, a difference before and after the reform can be observed. Second, enterprises in nonexperimental areas of the control group and those in the experimental area have heterogeneity, and intergroup differences exist (i.e., the grouping effect). Combining these two differences, the DID method can effectively identify the treatment effect before and after the implementation of policies.

The specific model of this study is set as follows:

$$ESG_{ijt}=\alpha_{0}+\alpha_{1}treat+\alpha_{2}time+\alpha_{3}did_{ijt}+\lambda X+\gamma_{i}+\mu_{t}+\varepsilon_{ijt}$$ (1)

In Eq (1), *i*, *j*, and *t* i indicate the listed enterprise, region, and founding year of the enterprise, respectively. The dependent variable *ESG*_*ijt*_ indicates the ESG disclosure scores of enterprises *i* in year *t*. Of the explanatory variables, *treat* is a dummy variable that can distinguish whether the GFPZ is set up in region *j*, and *time* is a dummy variable that distinguishes whether the GFPZ is set up in period *t*. *did*_*ijt*_ is the *treat*×*time* interaction term that is constructed in this paper. The estimated coefficient *α*_1_ reflects the net effect of policy implementation (i.e., the impact of GFPZ on corporate ESG performance), which is the focus of this paper. Table 1 illustrates the policy net-effect calculation process. Additionally, this paper introduces a series of control variables, represented by *X*. *γ*_*i*_ and *μ*_*t*_ indicate individual and time fixed effects, respectively; ε_ijt_ represents the random disturbance term. Specific definitions and measures of important variables are shown below.

**Table 1:** Description of the policy net-effect calculation process.

	Before the Policy	After the Policy	Difference
Treatment group	*α*_0_+*α*_1_	*α*_0_+*α*_1+_*α*_2_+*α*_3_	*α*_2_+*α*_3_
Control group	*α*_0_	*α*_0_+*α*_2_	*α*_2_
Difference	*α*_1_	*α*_1_+*α*_3_	*α*_3_ (DID)

### 4.2 Significant variables and their measures

#### 4.2.1 Dependent variable

ESG disclosure scores cover a variety of issues pertaining to environmental and social responsibility, and they serve as an important standard for measuring the sustainable development ability of enterprises. The degree of corporate social responsibility information disclosure can effectively reflect its performance, and the two are positively correlated according to Yu’s study [59]. Therefore, this paper selects the Bloomberg ESG disclosure scores as a proxy variable to measure the ESG performance of enterprises. This index provides a comprehensive evaluation of the performance of corporate social responsibility based on the Global Reporting Initiative combined with the company’s annual declaration documents, corporate social responsibility report, corporate website, questionnaires, media reports, and other public disclosure data. This index also provides a quantitative criterion for judging the enterprise ESG performance. This paper uses logarithmic conversion of the original data to diminish the heteroscedasticity problem.

#### 4.2.2 Explanatory variables

Two virtual variables are constructed in the model in this paper. One is the policy dummy variable "treat" in the pilot area, which assigns a value of 1 to enterprises located in the pilot cities (i.e., the treatment group) and a value of 0 to other enterprises located in non-pilot cities (i.e., the control group). The other is the time dummy variable, which assigns 1 to the year after the implementation of the policy (2017 and later), and 0 to the year before the policy implementation.

#### 4.2.3 Control variables

Considering that the ESG performance of enterprises is also affected by many factors, to reduce the endogenous bias caused by omitted variables, in this paper, we select a series of control variables at the firm level and the local municipality level, based on a combination of our own hypotheses and referencing previous relevant studies. Of them, the enterprise level includes the cash holding, which reflects the financial security and risk resistance of the enterprise. The city level includes financial autonomy, which reflects the level of financial revenue. Table 2 demonstrates the specific meanings of the control variables and their formulas, and Table 3 shows the descriptive statistics of the main variables.

**Table 2:** Meaning of control variables and calculation methods.

	Variable Name	Variable Name	Calculation method
Enterprise Level	*lnage*	Enterprise age	ln (year—listing year)
	*size*	Enterprise size	ln (total assets)
	*tang*	Asset structure	(Net value of fixed assets + net amount of inventory) / total assets
	*cash*	Cash holdings	(Monetary capitals + trading financial assets) / total assets
	*tl*	Asset-liability ratio	total liabilities / total assets
	*invt*	Investment spending rate	Cash paid to acquisitions of fixed assets, intangible assets, and other long-term assets / total assets
	*roa*	Net profit ratio of total assets	Net profit / total assets
	*tobin*	Tobin’ Q	The company’s market value / asset replacement cost
	*lnExecu*	Management scale	ln (number of directors, supervisors, and senior officers)
	*top*5 *HHI*	Concentration degree of shareholder shareholding ratio	The HHI index of the top five shareholders
City level	*lgdp*	GDP per capita	GDP/total population
	fa	Fiscal autonomy	General local budget revenue/general local budget expenditure
	to	Trade openness	Total industrial output value of foreign-invested enterprises/GDP
	*el*	Levels of urban human capital(Education level)	ln (number of students enrolled in general higher education institutions)
	sa	Urban savings levels	Year-end urban and rural savings balance/total population
	ue	Urban unemployment	ln (number of urban registered unemployed persons at the end of the year)
	is	Industrial structure	ln (value added in secondary sector)/ln (GDP)

**Table 3:** Descriptive statistics of variables.

	Variable Name	Sample Number	Mean	Std. Dev.	Min	Max
Dependent variable	*ESG*	3180	3.129	0.255	2.207	4.107
Enterprise -level control variables	*lnage*	3180	2.585	0.523	0.693	3.296
	*size*	3180	23.29	1.445	20.44	27
	*tang*	3180	0.426	0.164	0.0537	0.954
	*cash*	3180	0.163	0.117	0.00231	0.810
	*tl*	3180	0.484	0.190	0.0644	0.886
	*invt*	3179	0.504	0.202	0.0610	0.891
	*roa*	3180	0.0501	0.0410	0.00219	0.199
	*tobin*	3141	0.0412	0.0571	-0.190	0.211
	*lnExecu*	2860	1.751	1.013	0.812	6.396
	*top*5 *HHI*	3157	2.956	0.205	2.398	3.638
City-level control variables	*lgdp*	2843	5.964	3.643	1.247	16.40
	*fa*	2843	0.484	0.229	0.0882	0.961
	*to*	3180	3.129	0.255	2.207	4.107
	*el*	3180	2.585	0.523	0.693	3.296
	*sa*	3180	23.29	1.445	20.44	27
	*ue*	3180	0.426	0.164	0.0537	0.954
	*is*	3180	0.163	0.117	0.00231	0.810

### 4.3 Sample selection and data description

It can be seen from the above analysis that, at present, China’s attention and requirements on corporate ESG disclosure are mainly focused on listed enterprises, so this paper defines the scope of the research sample as A-share industrial listed enterprises in Shanghai and Shenzhen. The time range is chosen as 2010–2020. Additionally, the sample values of ST and ST* enterprises, delisted enterprises, and enterprises with both A-share and B-share issuance are excluded from the specific empirical test. Panel data for prefecture level cities are mainly obtained from the China City Statistical Yearbook and the China County Statistical Yearbook. We winsorize continuous variables and some of the variables are also normalized using logarithmic conversions and linear transformations, as required, in order to mitigate the effect of outliers on empirical results.

## 5. Empirical results

### 5.1 Baseline regression

Table 4 shows the regression results of the establishment of GFPZ on ESG disclosure scores in 2017. Column (1) shows the estimation results when only the time fixed effect and the individual fixed effect are controlled, without adding control variables. As shown in Column (1), the regression coefficient of the interaction term explanatory variable is 0.0820, which is significant at the 5% confidence level. This result indicates that the establishment of the GFPZ has significantly improved the ESG disclosure scores of enterprises in the region. Columns (2) and (3) introduce city-level and enterprise-level control variables on top of column (1) respectively, while column (4) adds both city-level and enterprise-level control variables, and the regression results show that the regression coefficients are significantly positive, in line with column (1). This result means that after the establishment of GFPZ, the ESG disclosure scores of enterprises in the region have increased significantly. That is, green-finance policies significantly improve the enterprises ESG performance, and this effect does not change significantly with the inclusion of control variables.

**Table 4:** Baseline regression results.

	(1) ESG	(2) ESG	(3) ESG	(4) ESG
did	0.0820** (0.0334)	0.102** (0.0404)	0.0964*** (0.0327)	0.136*** (0.0394)
City-level control variables	No	Yes	No	Yes
Enterprise -level control variables	No	No	Yes	Yes
Individual FE	Yes	Yes	Yes	Yes
Year FE	Yes	Yes	Yes	Yes
*R2*	0.254	0.239	0.295	0.286
Observations	3 180	2 594	2 803	2275

Notes

***, **, and * indicate that the regression results are significant at the 1%, 5%, and 10% levels, respectively. The following table uses the same process.

### 5.2 Robustness tests

#### 5.2.1 Parallel trend test: Dynamic effect analysis

The sample needs to meet the hypothetical premise of "parallel trend" when applying the DID model to evaluate a policy effect. This means that the ESG disclosure scores of enterprises in the pilot and non-pilot regions should maintain a consistent change trend. In this paper, we adopt the event study method to test for annual dynamic effects to determine whether this hypothesis is met. The following model is constructed:

$$ESG_{ijt}=\beta_{0}+\sum_{t=-M}^{N}\beta_{t}treat\times time_{t}+\lambda X+\gamma_{i}+\mu_{t}+\varepsilon_{ijt}$$ (2)

**Fig 2:**
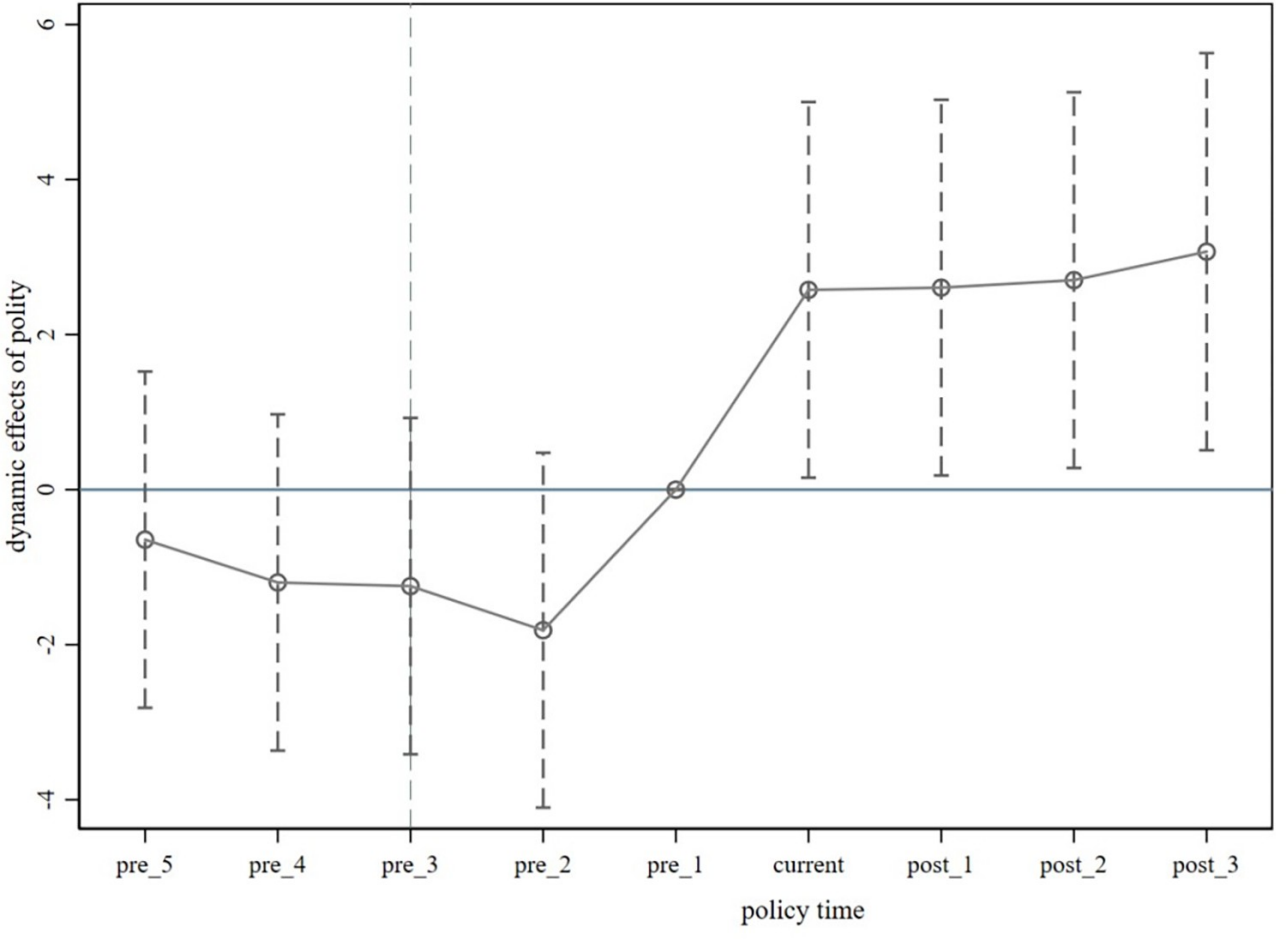
Parallel trend test. Notes: “current” represents the current policy period (2017), "pre_1" and "pre_2" represent 1 year and 2 years before the policy, respectively, and "post_1" and "post_2" represent 1 year and 2 years after the policy, respectively.

The essence of this method is to take the previous period of the policy (2016) as the benchmark group; then, construct cross-multiplication terms for the dummy variable (*time*_*t*_) and the treatment group dummy variable (*treat*) in each period, and the significance of a series of interaction items is examined. The lack of a significant difference between interaction terms before policy implementation indicates that the DID model can be used. In Eq (2), ***M*** denotes pre-policy years, and ***N*** denotes post-policy years. *β*_*t*_ is a series of coefficient estimates, and other variables are as shown in Eq (1). As shown in **Fig 2**, the coefficients are not significant before the implementation of the policy, suggesting that the ESG disclosure scores of the enterprises in the pilot zone and the control group have a "parallel" trend of change before the implementation of the policy, and that there is no significant difference.

#### 5.2.2 Placebo test: Random assignment of treatment groups

Considering that the above results could be caused by other random factors, we perform a placebo test by randomizing the treatment group [60]. The logic of a placebo test is to find the error variable "ftreat" that will not affect the result variable in theory to replace the real "*treat*." The randomly selected sample is first defined as the treatment group and otherwise as the control group. The interaction terms composed of the randomly sampled "*ftreat*" are then regressed on whether the kernel density maps of the observed coefficient or the observed values are centrally distributed around 0. Because the error variable "*ftreat*" is generated by random sampling, the actual policy effect on the enterprise ESG disclosure scores is 0. Therefore, if the estimated regression coefficient is not 0, this result suggests that the regression results are biased and that unobserved features affect the estimated results.

**Fig 3:**
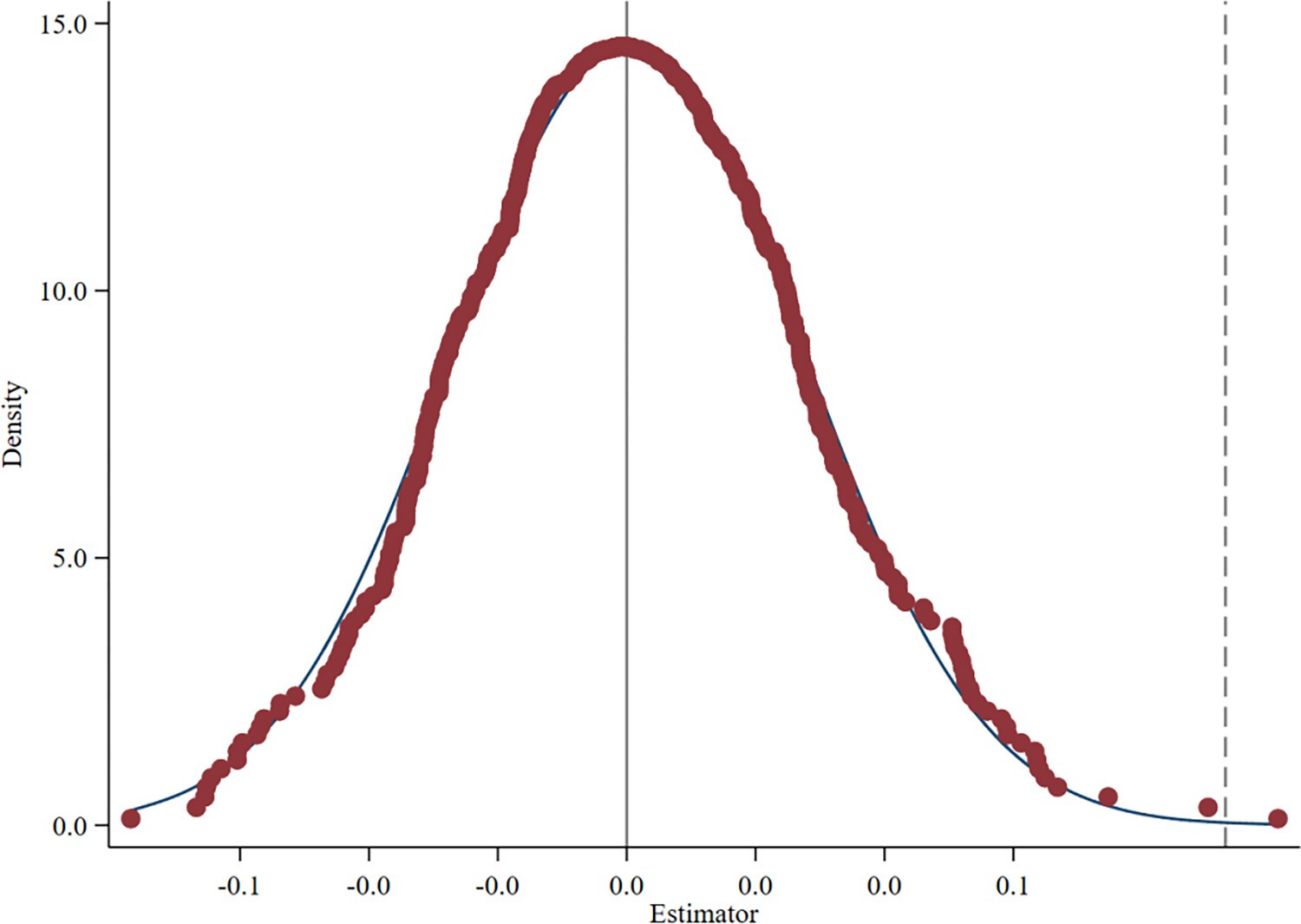
Distribution map of the kernel density of estimated coefficients.

**Fig 4:**
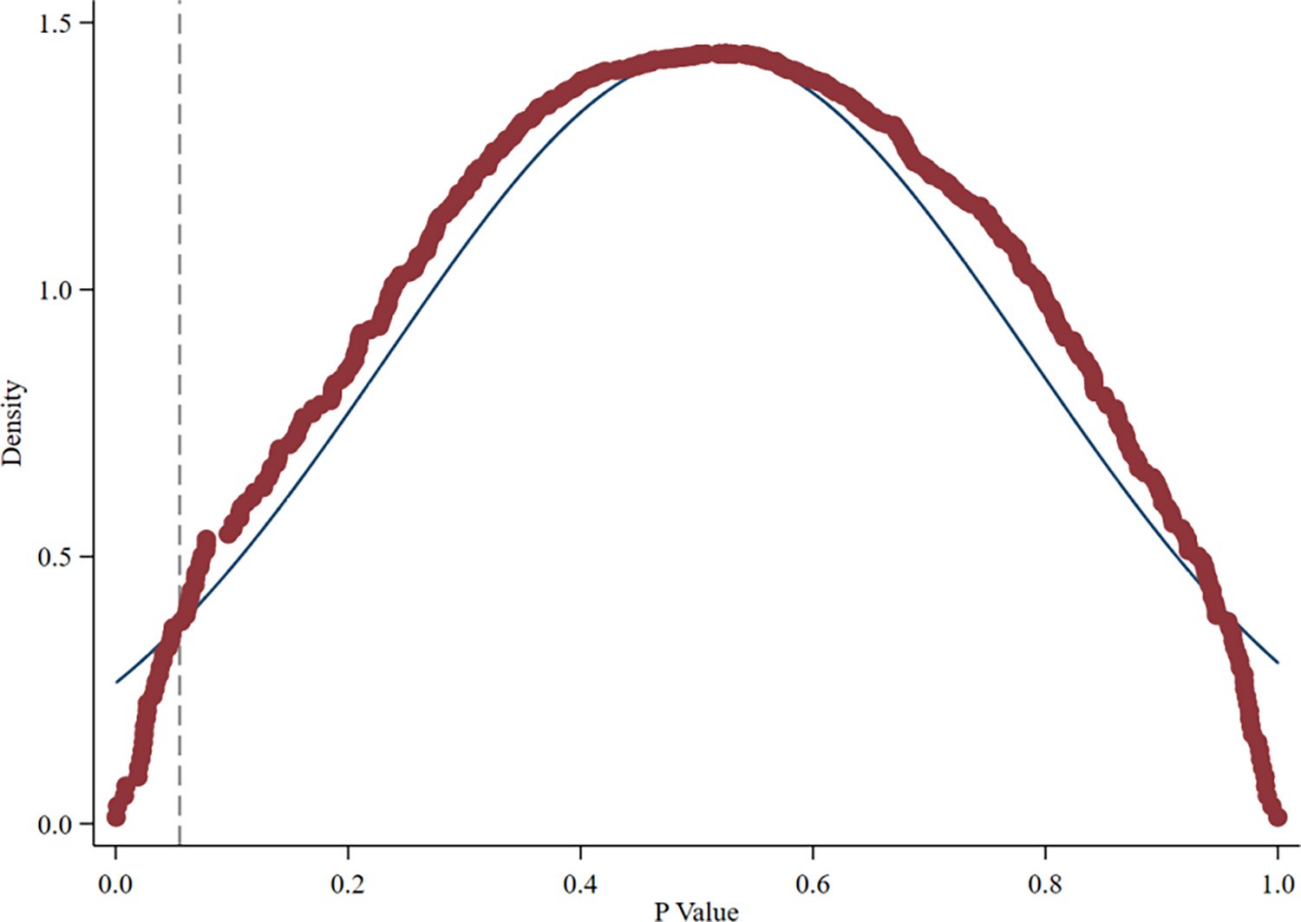
Distribution map of the P values of estimated coefficients.

**Fig 3** depicts the kernel density distribution of the estimates of interaction term coefficients across 500 regressions. The estimated coefficient of the interaction term is concentrated around 0, and the dashed line shows the estimated result of the baseline regression above, which clearly shows outliers in this placebo test (that differ significantly from 0). **Fig 4** presents the scatter distribution of P values of interaction term coefficients across 500 regressions. Apparently, most scatter points in Fig 4 are located on the right side of the dotted line with P = 0.05 (5% confidence level), indicating that these regression coefficients are not significant at least at the 5% confidence level. In summary, most estimated coefficients of interaction terms are not significant in the randomly generated treatment group, meaning that the policy effect of GFPZ policy on the enterprises ESG disclosure scores is not affected by other unobserved factors.

#### 5.2.3 Replace the dependent variable: CSR score from Hexun

Considering the diversity of enterprise ESG performance measures, this paper tests the robustness of the regression by re-selecting the dependent-variable measures. The CSR scoring system from the Hexun Database is selected as the new dependent variable (CSR). The score not only covers a wide range of samples but also includes five dimensions of indicators related to corporate ESG performance, such as consumer fairness responsibility, which is the scientific and authoritative index of enterprise ESG performance and is widely used by scholars [61]. In this paper, the raw score is log-transformed. The results in columns (1) and (2) of Table 5 show that the regression coefficients with Hexun CSR scores as the dependent variable are significantly positive, consistent with the baseline regression results. This result proves that the empirical results do not vary with the dependent-variable measurement method.

**Table 5:** Robustness tests results.

	Replace Dependent Variable		Reduce the Sample Range	
	(1) CSR	(2) CSR	(3) ESG	(4) ESG
did	0.294** (0.140)	0.305** (0.141)	0.0680** (0.034)	0.107*** (0.0375)
City-level control variables	No	Yes	No	Yes
Enterprise -level control variables	No	Yes	No	Yes
Individual FE	Yes	Yes	Yes	Yes
Year FE	Yes	Yes	Yes	Yes
*R2*	0.333	0.401	0.264	0.311
Observations	2 780	2 493	1 901	1 701

#### 5.2.4 Control of other policy interference: Reduce the sample range

Considering that in recent years, China has formulated other relevant policies in the area of green finance. For example, the Green Credit Guidelines were promulgated in 2012, providing a clear direction for the development of green-finance credit. To avoid the possible impact of green credit policies in 2012 on the effect test of baseline regression, in this paper, the sample selection range is shortened to 2014–2020 to identify the net effect of GFPZ policies. The empirical results are presented in Table 5. As shown in Columns (3) and (4), the regression coefficients of interaction term explanatory variables are significantly positive at the 1% confidence level, which is consistent with the baseline regression results, indicating the robustness of our conclusions.

### 5.3 Mechanism analysis

#### 5.3.1 GFPZ policy improves enterprises’ external financing capability

At present, the capital source of enterprises still depends on external financing, among which short-term borrowing is an important source. Short-term borrowing is mainly used to compensate for the short-term capital turnover needs of enterprises. Compared with long-term borrowing, short-term borrowing is more susceptible to external uncertainties (e.g., policy change). Therefore, short-term enterprise borrowing is selected as the dependent variable (lndd). This paper uses the logarithmic conversion of the original data.

Columns (1) and (2) of Table 6 show that the regression coefficients are significantly positive at the 1% confidence level, suggesting that the establishment of the GFPZ policy significantly increases the amount of short-term borrowing and expands the scale of external financing. In fact, because of the brief repayment period of short-term borrowing, creditors impose high requirements on the capital liquidity and solvency of borrowing enterprises; therefore, they need to assess more financial information of the enterprises. By setting up information sharing platform such as disclosure of enterprises’ polluting behaviors, the GFPZ policy has effectively alleviated the information asymmetry between capital suppliers and enterprises, thus promoting the effective allocation of capital.

**Table 6:** Results of mechanism analysis.

	(1) *lndd*	(2) *lndd*	(3) *lnzl*	(4) *lnzl*
did	0.974*** (0.241)	0.653*** (0.225)	0.523*** (0.199)	0.611*** (0.231)
City-level control variables	No	Yes	No	Yes
Enterprise -level control variables	No	Yes	No	Yes
Individual FE	Yes	Yes	Yes	Yes
Year FE	Yes	Yes	Yes	Yes
*R2*	0.075	0.349	0.111	0.148
Observations	2 482	1 958	2 348	1 655

#### 5.3.2 GFPZ policy promote enterprises’ green-technology innovation

In this paper, the number of green patent applications is used as the dependent variable (lnzl), and the raw data are subjected to natural logarithmic treatment to avoid the problem of right-skewed distribution of the data. Columns (3) and (4) of Table 6 show that the regression coefficients are significantly positive at the 1% confidence level, suggesting that the policy effectively promotes the green-technology innovation of urban enterprises located in the pilot zone.

In fact, green-technology R&D requires more funding and for a longer period than general innovation. Faced with the high technology development costs, managers who follow the rational hypothesis often lose the will to undertake green-technology development activities. The establishment of GFPZ provides financial support for enterprises in the region to invest in green production technology by encouraging the development of green credit and establishing a green-finance risk-prevention mechanism. The capital orienting the environmental protection projects transmits the signal of profitable green-technology innovation and enhances the ability and motivation of enterprises to achieve long-term sustainable development through green-technology innovation.

### 5.4 Heterogeneity analysis

The policy impact affects enterprises with different endowments and characteristics to varying degrees. Exploring the different responses of enterprises to policy impact is helpful in gaining a comprehension grasp of the effect of policies and in providing an experience reference for improving and implementing the policy in the next step. Therefore, this paper further assesses the heterogeneity of impacts.

#### 5.4.1 Heterogeneity of the nature of enterprise equity

Table 7 demonstrates the empirical results of the impact of the GFPZ policy on enterprises with different equities. The establishment of the GFPZ has significantly improved the ESG disclosure scores of SOEs, but the results are not significant for the sample of non-SOEs. The main reason could be that under the special institutional background of China, SOEs have both political and public attributes. The government has put forward higher requirements for them by enhancing social and public interests, and non-SOEs usually mainly aim to make profit. Most non-SOEs enterprises do not have sufficient financial and material resources to support ESG practice.

**Table 7:** Empirical results of equity nature heterogeneity.

	State-owned Enterprises ESG		Non-state-owned Enterprises ESG	
did	0.182*** (0.0490)	0.258*** (0.0545)	-0.0710 (0.0553)	-0.0055 (0.0539)
City-level control variables	No	Yes	No	Yes
Enterprise -level control variables	No	Yes	No	Yes
Individual FE	Yes	Yes	Yes	Yes
Year FE	Yes	Yes	Yes	Yes
*R2*	0.255	0.313	0.220	0.324
Observations	1 839	1 444	9 83	805

#### 5.4.2 Heterogeneity of the internal control quality of enterprises

Internal control is reflected in all of the aspects of enterprise production and operation, promotes enterprises to standardize organizational decision-making, and then acts on enterprise behavior. Therefore, this paper introduces the internal control disclosure composite index (lnic) to measure the quality of firms’ internal control, with data from the DIB database. The index includes five aspects, such as the business legal compliance, and it is supplemented and corrected according to the major defects of internal control, which can effectively reflect the internal control performance of the enterprise. Additionally, this paper adopts logarithmic processing of the original data.

Table 8 shows the heterogeneity impact of the GFPZ policy on enterprises with different internal control qualities. The GFPZ policy has a significant improvement effect on enterprises with better internal control quality but no significant effect on enterprises with lower quality. This is because high-quality internal control ability is the basis for corporate governance structure to play a role while helping regulate and supervise the behavior of managers, helping the management make more favorable decisions according to the future development form and their own business conditions, and providing a guarantee for enterprises to fulfill their environmental and social responsibilities.

**Table 8:** Heterogeneity of the internal control quality of enterprises.

	Enterprises with Low Internal Control Quality ESG		Enterprises with High Internal Control Quality ESG	
did	0.0122 (0.0443)	0.0623 (0.0500)	0.175*** (0.0610)	0.205*** (0.0647)
City-level control variables	No	Yes	No	Yes
Enterprise -level control variables	No	Yes	No	Yes
Individual FE	Yes	Yes	Yes	Yes
Year FE	Yes	Yes	Yes	Yes
*R2*	0.178	0.254	0.307	0.367
Observations	1 404	1 206	1 435	1 069

### 5.5 Further analysis

It has been shown that active corporate social responsibility contributes to a sustainable trinity of economic, environmental, and social standards. For this reason, this paper conjectures that promotion of GFPZ on enterprise ESG performance can improve the level of environmental governance in the region where the enterprise is located, with good environmental benefits. To test this conjecture, this paper constructs the following model:

$$EBI_{j}=\gamma_{0}+\gamma_{1}did_{jt}+\gamma_{2}treat+\gamma_{3}time+\lambda X^{'}+\varphi_{j}+\mu_{t}+\varepsilon_{jt}$$ (3)

The dependent variable (*EBI*_*j*_) is a composite index of environmental benefits constructed in this paper, which represents the level of environmental governance in the city *j* and is used to reflect the environmental benefits to the city of the GFPZ in stimulating corporate social responsibility. The index is constructed following the principle of focusing on the actual improvement effect of policies on the environment. Taking into account the current situation of various pollutant emissions in China and the availability of data, we select four single indicators: industrial sulfur dioxide removal, industrial soot removal, comprehensive utilization rate of general industrial solid waste and industrial wastewater discharge compliance, and the weighted linear sum method adopted by Shen in measuring the degree of environmental regulation [62], with the following process.

First, the individual indicators are standardized:

$${p^{'}}_{jm}=[p_{jm}-min(p_{m})]/[max(p_{m})-min(p_{m})]$$

where *p*_*jm*_ is the raw data of the single indicator of category *m* in city *j*, *max*(*p*_*m*_) and *min*(*p*_*m*_) are the maximum and minimum values of the single indicator of category m respectively, and *p*’_*jm*_ is the standardized value of the single indicator of category *m* in city *j*.

Then, taking into account the different levels of emissions of different pollutants within the same city and the differences in the weight of emissions of the same pollutant between different cities, in order to reflect more accurately the differences in the environmental improvement effect of each city, we calculate different weights for each individual indicator:

$$A_{jm}=\frac{p_{jm}}{\sum_{j}p_{jm}}/\frac{GDP_{j}}{\sum_{j}GDP_{j}}$$

where *A*_*jm*_ is the weight of the individual indicator of category *m* in city *j*, and *GDP*_*j*_ is the regional GDP of city *j* in that year.

Finally, based on the standardized values and weights *A*_*jm*_ of the indicators calculated above, a comprehensive index of environmental benefits *EBI*_*j*_ is constructed: $EBI_{j}=\sum_{m}^{4}A_{jm}{p^{'}}_{jm}/4$.

The results of columns (1)–(4) in Table 9 are consistent and all of them indicate that the regression coefficients are significantly positive, suggesting that the positive promotional impact of the GFPZ on the social responsibility performance of enterprises has significantly improved the environmental governance of the municipality where they are located, bringing good environmental benefits to the municipality.

**Table 9:** Empirical results for further analysis.

	(1) EBI	(2) EBI	(3) EBI	(4) EBI
did	0.332*** (0.0874)	0.249*** (0.0748)	0.475*** (0.176)	0.385*** (0.145)
City-level control variables	No	Yes	No	Yes
Enterprise -level control variables	No	No	Yes	Yes
Individual FE	Yes	Yes	Yes	Yes
Year FE	Yes	Yes	Yes	Yes
R2	0.055	0.075	0.066	0.093
Observations	2 787	2 787	2 443	2 443

## 6. Conclusions and implications

### 6.1 Discussion

This paper finds that green-finance policy firms have a positive effect on firms’ ESG performance. This finding is consistent with those of Huang and Lei [3], who point out that the environmental context in which firms are located, such as the policy system and financial resources, is a factor that affects the ability of firms to fulfill their social responsibilities. Moreover, green finance is a new type of policy tool with the dual characteristics of environmental regulation and financial constraints. Therefore, the findings of this paper provide a new theoretical perspective that clarifies the relationship between the institutional environment, financial resources, and ESG performance. In the mechanism analysis, this paper points out that GFPZ can improve firms’ external financing ability and green-technology innovation ability, which further enhances the long-term sustainability of firms. This result is consistent with Zhang [33], who argue that green finance has a positive incentive effect on corporate green innovation. However, Xu and Li argues that green finance is detrimental to corporate R&D investment and innovation output [34], contrary to the findings of this paper. In the heterogeneity analysis, this paper finds that green-finance policies significantly increase the ESG disclosure scores of SOEs, but the empirical results for the sample of non-SOEs are not significant. This observation, together with those of Xin, also suggests that financial-model innovation positively affects firms’ social responsibility performance, and this effect increases with the increase of firms’ equity concentration [63].

### 6.2 Conclusions and implications

This paper takes the establishment of GFPZ in 2017 as a quasi-natural experiment, and based on the data of a-share listed companies in Shanghai and Shenzhen from 2010 to 2020, this paper empirically examines the effect and mechanism of the GFPZ on the ESG performance of enterprises using the DID model. The following valuable findings are obtained: First, the GFPZ policy effectively promotes enterprises in the region to actively implement ESG practices. Second, green finance has a positive effect on enterprise ESG performance by improving external financing capacity and green-technology innovation. Third, there is heterogeneity in the impact of GFPZ policy on the ESG performance of firms with different equity natures and internal control levels. Specifically, the policy significantly improves the ESG disclosure score of SOEs and firms with higher quality of internal control. However, the empirical results are not significant for the sample of non-state-owned firms and firms with a lower quality of internal control. Fourth, this paper finds that green finance promotes enterprises to actively undertake social responsibility, which can further improve the level of environmental governance in the region where it is located and has good environmental benefits.

Based on the above theoretical analysis and empirical tests, the following recommendations are provided: First, the supply-side structural reform of finance should be promoted in a targeted way to alleviate the asymmetry of the effect of policies among heterogeneous enterprises. As the influence of GFPZ policies on different enterprises is heterogeneous, the design of the system should take into account the different endowment characteristics of enterprises and different pilot zones. Moreover, the government should take advantage of the situation and adapt to local conditions, further optimize the institutional design, supplement incentive measures, create a better and fairer external financing environment and institutional guarantee, and help financial resources achieve better allocation. Second, the GFPZ policy should be implemented, and the supporting measures for policy implementation should be improved. Government departments can help enterprises with green-technology innovation and prompt enterprises to better carry out ESG practice. Enterprise ESG performance can also be improved by increasing investment in infrastructure construction, formulating policies for personnel training and introduction, and improving IPR protection mechanisms for green-technology innovation.

There are still certain limitations of this study that deserve further attention. First, green finance includes different types of policy instruments, such as green credit, green insurance, and so on. Therefore, in future studies, the influence of different green-finance instruments on enterprise ESG performance can be explored further. Second, the Bloomberg ESG disclosure score used in this paper mainly focuses on the amount of ESG data that is publicly disclosed by enterprises. With the continuous advancement of academic research, the comprehensive evaluation model for the performance of enterprises’ ESG will be further perfected, and proxy variables by other measures can be chosen to more objectively and comprehensively understand corporate ESG practices. Third, in this study, enterprise ESG performance are simplified into one-dimensional variables, but the impact of green finance on enterprise ESG performance is a complex process. Therefore, in future studies, the influence of green finance on the multidimensional characteristics of enterprise ESG performance can be explored more comprehensively, and the internal influence mechanism of green finance and enterprise ESG performance can be understood in greater depth.
